# Oral Elastofibromatous Lesion on the Tip of the Tongue: A Case Report and a Literature Review

**DOI:** 10.7759/cureus.65430

**Published:** 2024-07-26

**Authors:** Makoto Toida, Itsuki Hayashi, Aina Yamaguchi, Hironobu Naiki

**Affiliations:** 1 Department of Oral and Maxillofacial Surgery, Sugita Genpaku Memorial Obama Public Hospital, Obama, JPN; 2 Department of Oral and Maxillofacial Surgery, Gifu University, Gifu, JPN; 3 Department of Diagnostic Pathology, Sugita Genpaku Memorial Obama Public Hospital, Obama, JPN; 4 Department of Molecular Pathology, University of Fukui, Fukui, JPN

**Keywords:** case report, elastofibroma, elastofibromatous change, elastofibromatous lesion, tongue, oral

## Abstract

Oral elastofibromatous lesions (OEFLs) are rare benign tumor-like lesions of the oral cavity, which are characterized by marked hyperplasia of elastic fibers. A case of OEFL on the tip of the tongue of an 83-year-old male is presented. The lesion was a painless, yellowish-white, smooth-surfaced, elastic-soft, well-demarcated, slightly flattened, round plaque-like mucosal lesion, measuring about 4 x 3 mm. The lesion was excised under local anesthesia with the clinical diagnosis of an irritation fibroma and a benign tumor including a lipoma, and diagnosed histopathologically as an OEFL. Four years and nine months after the surgery, no abnormality was observed at the surgical site, and there were no signs of recurrence. In addition to a report of the case, the clinical and histopathological features of 14 similar cases reported in the English literature were reviewed.

## Introduction

Elastofibroma dorsi (EFd), which was first described as an uncommon benign soft-tissue pseudotumor in 1961, typically occurs in the subscapular region of middle-aged or older individuals [1]. Recent cytogenetic and molecular genetic studies suggest that EFd is a possible neoplastic process [1], and the 2020 World Health Organization Classification of Tumours of Soft Tissue and Bone [2] classified the lesion as the fibroblastic/myofibroblastic tumor group, although it has been thought to be a reactive lesion resulting from chronic frictional and physical irritation or repeated minor trauma [1]; the pathogenesis of EFd is still unclear [1]. The elastofibroma (EF) occurs most frequently in the subscapular or infrascapular region as EFd [1,2], and similar lesions rarely occur in other locations including hands, feet, thighs, gastrointestinal tract, and even more rarely in the oral cavity; only a dozen or so cases of such oral lesions have been reported in the English-language literature [3-10]. These oral lesions have been reported under various names, including oral elastofibromatous lesion (OEFL) [7,9] and oral elastofibromatous change (OEFC) [5,6], aside from oral elastofibroma (OEF) [3,4,8], because it is unclear whether these oral lesions are true neoplasm or not. Moreover, the OEFL/OEFC/OEF are almost exclusively composed of elastic fibers, rather than showing an equal proportion of collagen and elastic fibers [7]; these oral lesions resemble an elastosis rather than the typical histopathologic features usually seen in EFd [7]. Thus, it is suggested that these oral lesions may not represent a true analogous lesion of the EFd and/or a true oral counterpart of the EFd. In this report, we tentatively choose the term OEFL to represent these oral lesions as a whole, which go by various terms as described above. The OEFL, like similar extraoral lesions including EFd, generally has a good prognosis, but differential diagnosis is important because it can be misdiagnosed clinically as malignant or precancerous lesions. We report a case of OEFL occurring at the tip of the tongue and add a literature review.

## Case presentation

**Figure 1:**
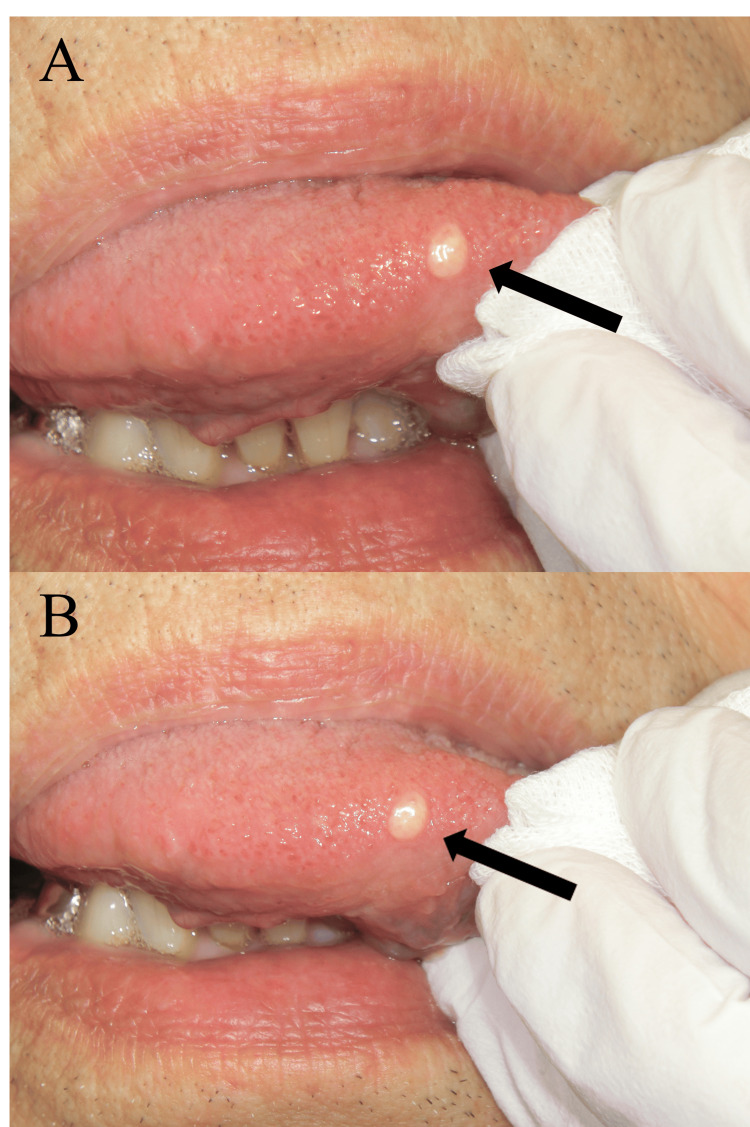
Clinical photographs of the lesion (arrow) at the time of the first visit (A) and two years and three months after the first visit (B). There is no significant difference in the shape or size of the lesion between (A) and (B).

In July 2017, an 83-year-old male visited our department for dental caries treatment and periodontal disease management. There was no history of systemic disease. On the day of the initial visit, an intraoral examination revealed a painless, yellowish-white, smooth-surfaced, elastic-soft, well-demarcated, slightly flattened, round plaque-like mucosal lesion, measuring about 4 x 3 mm, on the right margin of the tip of the tongue (Figure 1A). There was no specific history of trauma to the region. Moreover, there was no history of irritant use or ingestion, such as smoking, alcohol consumption, herbal medicine, oral antiseptics, mouthwash, or previous use of oral topical treatments. Surgery was recommended, but the patient refused surgery because the lesion had been present for more than 40 years and it had not changed significantly.

The patient continued to visit our department for general dental treatment only. Although there was no significant change in the lesion in shape or size (Figure 1B), the patient requested surgery about two years and three months after the initial visit. In October 2019, the lesion was excised under local anesthesia with the clinical diagnosis of an irritation fibroma and/or a benign tumor including a lipoma. The lesion was excised wedge-shaped with about 10 x 7 mm spindle-shaped incision that included the normal mucosa surrounding the lesion.

**Figure 2:**
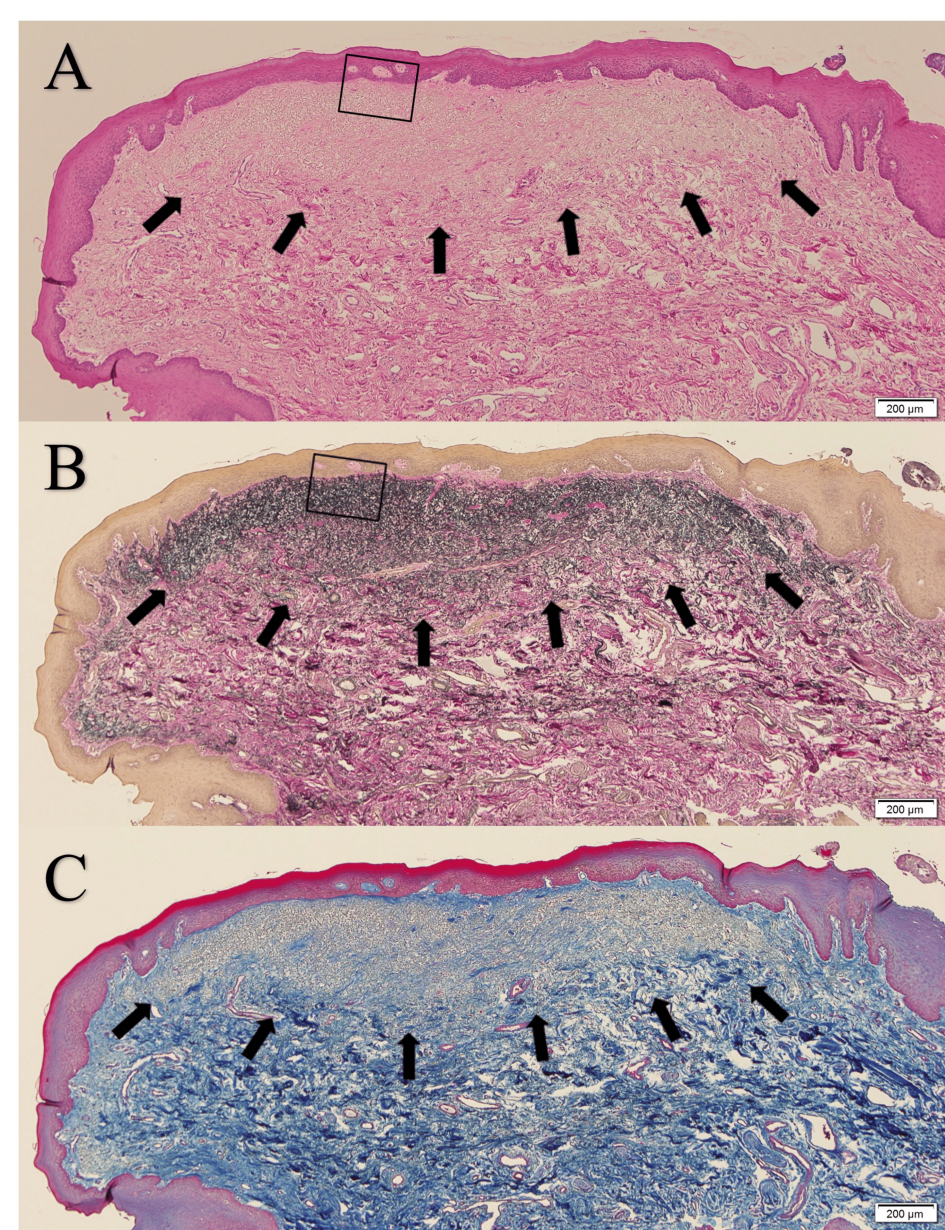
Microscopic view of the lesion at low magnification (A) Accumulation of delicate eosinophilic fibers (arrows) are seen beneath the acanthotic keratinized stratified squamous epithelium (hematoxylin and eosin stain, scale bar = 200 μm); (B and C) the delicate fibers composing the lesion (arrows) are stained black-purple with Elastica van Gieson stain (B), but are not stained dark blue with azocarmine-aniline blue stain (C) (scale bar = 200 μm).

**Figure 3:**
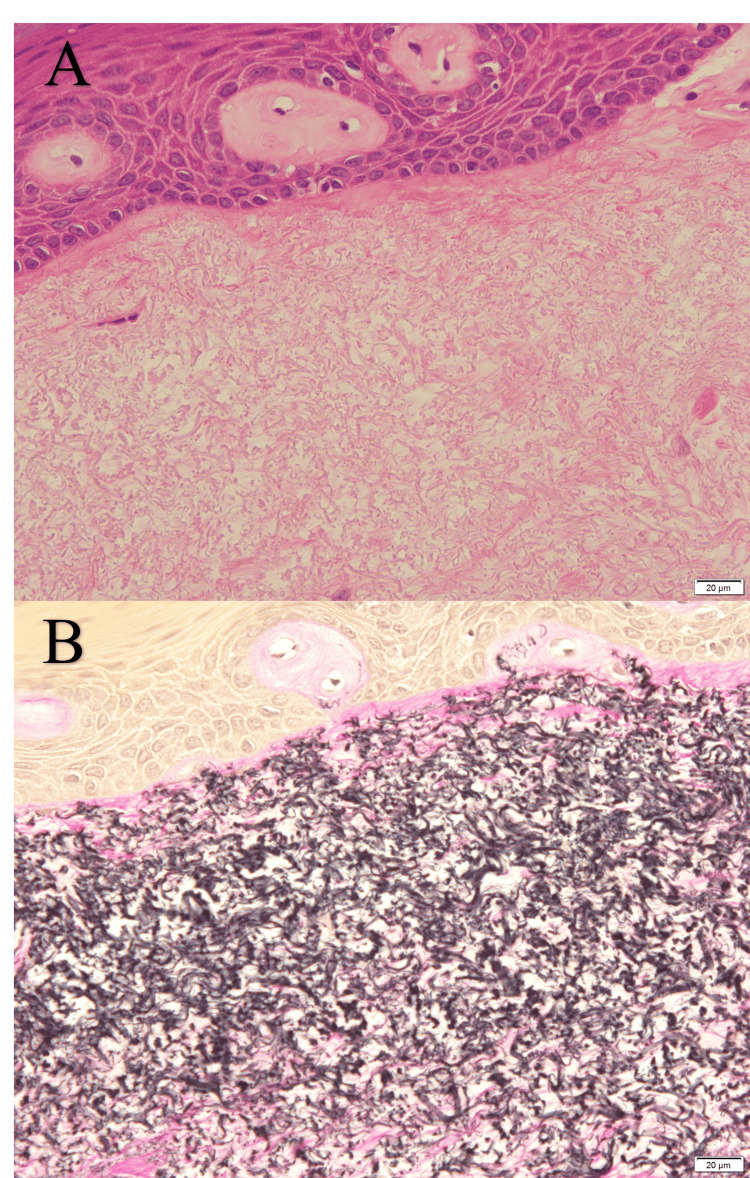
Micrographs of the lesion at high magnification in the squared areas in Figures 2A-2B. The delicate eosinophilic fibers comprising the lesion (A) are stained black-purple with Elastica van Gieson stain (B) (scale bar = 20 μm).

Histopathologically, the lesion was composed of delicate eosinophilic fibers, which were densely accumulated (Figure 2A and Figure 3A) beneath the acanthotic keratinized stratified squamous epithelium; the delicate fibers formed an uncapsulated nodule-like lesion with a somewhat indistinct border with the surrounding fibrous connective tissue (Figure 2A). The delicate fibers were stained black-purple by Elastica van Gieson stain (Figure 2B and Figure 3B), were negative for Periodic acid-Schiff stain, and were identified as elastic fibers. Collagen fibers, that should be stained red with Elastica van Gieson stain and blue with azocarmine-aniline blue stain, were sparse within the lesion (Figures 2B-2C and Figure 3B). Thus, the lesion consisted almost exclusively of elastic fibers, whereas the fibrous connective tissue surrounding the lesion showed marked and extensive bundles of collagen fibers and a paucity of elastic fibers (Figures 2B-2C and Figure 3B). Based on these findings, the lesion was diagnosed as OEFL.

Healing was excellent at the time of suture removal one week after surgery. The patient continued to visit our department regularly for periodontal disease management. Four years and nine months after the surgery, no abnormality was observed at the surgical site, and there were no signs of recurrence.

## Discussion

Sixteen cases of OEFL and similar lesions have been reported in nine English-language references [3-11]. Of these, one of the three cases reported by Tosios et al. [5] was a simple hyperelastosis of the oral mucosa, and one by Silveira et al. [11] was extensive elastofibromatous changes in the intraoral epidermoid cyst. These two cases differ significantly from the other cases in their clinical presentation. After excluding these two cases, a total of 15 cases (Table 1), consisting of 14 cases from eight references [3-10] and the present case, were examined for clinicopathological features, differential diagnosis, treatment, and prognosis, with the following results.

**Table 1:** Clinicopathological aspects of 14 reported cases and the present case of elastofibromatous lesion/change F: female; M: male; NC: not clear; NER: no evidence of recurrence; NS: not stated; SCC: squamous cell carcinoma

Authors	Age	Sex	Location	Duration	Clinical appearance	Size	Clinical diagnosis	Previous history of trauma	Treatment	Follow-up	Histopathological diagnosis
Potter et al. (2004) [3]	56	F	Floor of the mouth	Less than 6 months	White, well-circumscribed submucosal mass, asymptomatic	0.4 x 0.3 cm	Benign soft tissue neoplasm, lymphoid aggregate, minor salivary duct cyst, and scar	Trauma, 52 years ago	Surgical excision	2 years, NER	Elastofibroma
Manchandu et al. (2008) [4]	71	M	Floor of the mouth	Less than 2 years	White, firm, smooth-surfaced, painless nodular mass	6 mm	Recurrent SCC	Surgery and radiotherapy for SCC of the tongue, 2 years ago	Surgical excision	14 months, NER	Elastofibroma
Tosios et al. (2010) [5]	76	F	Floor of the mouth	NS	Small, flat, white area	NS	NS	NS	Surgical excision	NS	Elastofibromatous change
Tosios et al. (2010) [5]	98	F	Alveolar mucosa close to the floor of the mouth	NS	Leukoplakia-like	NS	Leukoplakia	SCC of the floor of the mouth	Surgical excision	NS	Elastofibromatous change
Nonaka et al. (2010) [6]	55	M	Soft palate	Less than 6 months	Whitish plaque, asymptomatic	1.0 cm	Leukoplakia	Orotracheal intubation, 1 year ago	Excisional biopsy	8 months, NER	Elastofibromatous change
Darling et al. (2011) [7]	33	M	Palate	NS	Asymptomatic, non-ulcerated lesion	2 x 2 mm	Fibroma	NC	Surgical excision	1 year, NER	Elastofibromatous lesion
Darling et al. (2011) [7]	43	M	Palate	NS	Asymptomatic, yellow growth	3 x 2 x 1 mm	Condyloma	NC	Surgical excision	9 years, NER	Elastofibromatous lesion
Darling et al. (2011) [7]	50	M	Floor of the mouth	NS	Leukoplakia-like	7 x 3 x 2 mm	Hyperkeratosis	NC	Surgical excision	3 years, NER	Elastofibromatous lesion
Darling et al. (2011) [7]	76	F	Lower lip	NS	Tan and white ellipse of mucosa	7 x 4 x 2 mm	Recurrent SCC	SCC at the site that had been treated with radiation	Surgical excision	10 years, NER	Elastofibromatous lesion
Darling et al. (2011) [7]	75	M	Tongue	NS	White nodule	5 x 4 x 4 mm	Fibroma	NC	Surgical excision	6 months, NER	Elastofibromatous lesion
Daley and Darling (2011) [8]	62	M	Hard palate	NS	Asymptomatic, exophytic, smooth-surfaced, pink module	3 mm	Fibroma	No specific history of trauma	Surgical excision	NS	Elastofibroma
Silva et al. (2020) [9]	63	F	Buccal mucosa	NS	Asymptomatic, localized, slightly rlevated whitish lesion	10 x 8 mm	Leukoplakia	No specific history of trauma or surgery in the lesion area	Biopsy	NS	Elastofibromatous lesion
Silva et al. (2020) [9]	76	M	Inferior vestibular fornix	6 months	Solitary, asymptomatic white lesion, with a round to oval morphology and smooth surface	1 cm	Lichen sclerosus et atrophicus	Soft tissue injury, 6 months ago; using total dentures for 48 years	Biopsy	NS	Elastofibromatous lesion
Ogawa et al. (2022) [10]	72	F	Posterior maxillary alveolar mucosa	NC	Round white-colored, smooth-surfaced, solitary firm papule	3 mm	Lipoma or submucosal abscess	NC	Excisional biopsy	4 months, NER	Oral focal submucous elastofibromatous papule
Present case	83	M	Right tip of the tongue	40 years or longer	Painless, yellowish-white, smooth-surfaced, elastic-soft, well-demarcated, slightly flattened, round plaque-like mucosal lesion	4 x 3 mm	Irritation fibroma or benign tumor including lipoma	No specific history of trauma or surgery in the lesion area	Surgical excision	4 years 9 months, NER	Elastofibromatous lesion

The age of the patient ranged from 33 to 98 years, with a mean age of 65.9 years. Nine patients were male and six were female, with male cases being 1.5 times more common than female cases. The lesions were located on the floor of the mouth (n = 4), the palatal mucosa (n = 4), the alveolar mucosa (n = 2), the tongue (n = 2), the buccal mucosa (n = 1), the inferior vestibular fornix (n = 1), and the lower lip (n = 1), with no cases of bilateral lesions. Duration of lesions was described in only five cases, with three cases lasting less than six months and one case lasting less than two years; no other case was found to have a long duration of more than 40 years as in the present case. In the present case, moreover, there were no significant changes in the shape or size of the lesion during the two years and three months between the initial examination and surgery. The lesions were all less than 1 cm in size, except for two lesions for which no size was noted. Clinical diagnoses varied as follows: fibroma/irritation fibroma (n = 4), leukoplakia (n = 3), benign tumor/benign soft tissue neoplasm (n = 2), lipoma (n = 2), recurrent squamous cell carcinoma (n = 2), condyloma (n = 1), hyperkeratosis (n = 1), lichen sclerosus et atrophicus (n = 1), lymphoid aggregate (n = 1), minor salivary duct cyst (n = 1), scar tissue (n = 1), and submucosal abscess (n = 1). In some cases, more than one clinical diagnosis was listed in a single case, and all of them were listed here; in one case, clinical diagnosis was not noted. There was a clear or suspected history of trauma in six cases, three of which had a history of squamous cell carcinoma at the relevant site of the lesion, and two of which had a clear history of treatment for squamous cell carcinoma. There was no specific history of trauma in three cases; the history was unknown in five cases and was not stated in one case. The treatment modalities were surgical excision in 11 cases and biopsy in four cases, in three of which were excisional biopsy. Postoperative follow-up duration was four months to 10 years in all 10 cases, excluding five cases in which no postoperative follow-up was noted, and there was no recurrence in any of the 10 cases. Pathological diagnoses were elastofibromatous lesion (n = 8), elastofibromatous change (n = 3), EF (n = 3), and elastofibromatous papule (n = 1).

Because OEFL is a poorly known lesion with no distinctive clinical features, it may have been overlooked and misdiagnosed as other benign tumors or tumor-like lesions, such as irritation/traumatic fibromas, and there have been very few reports of this disease. Although the prognosis for this lesion is good, with few recurrences after simple resection, it may also be misdiagnosed preoperatively as malignant or precancerous lesions [4-7,9]. Therefore, the OEFL should be included in the differential diagnosis when the lesions with such clinicopathologic features are encountered.

Histopathologically, it has been reported that the proportion of fibrous components in EFd is almost equal between elastic and collagenous fibers [1,2], but the amount of elastic fibers tends to be much larger than that of collagenous fibers in oral cases [7]. As mentioned by Darling et al. [7], the entire lesion of typical OEFL is almost exclusively composed of markedly increased elastic fibers and lacks the marked bundles of collagen fibers typical of usual oral irritation/traumatic fibromas, and it is the same in their cases as well as in the present case.

Family history, clonal aberrations, and chromosomal instability suggest that EFd is a neoplastic process [1,2], although it has been suggested that EFd represents a reactive lesion resulting from chronic frictional and physical irritation or repeated microtrauma rather than a true neoplasm [1]; the pathogenesis is not clear [1]. On the other hand, the involvement of chronic irritation and/or trauma has been reported relatively frequently in oral lesions. The presence of cases such as the present case, which have not changed in shape or size over a long period of time over 40 years, also raises the question of its neoplastic nature. At this time, it is unclear not only whether the oral lesion is a true neoplasm, but also whether it is an analogous lesion in the oral cavity of EFd.

Recently, Rodrigues et al. [12] reviewed their 95 cases that had been diagnosed as oral fibroma on specimens stained by hematoxylin and eosin, and reclassified 56 cases as EF, 21 as EF-like lesion (EF-like structure), and 18 as oral fibroma, according to their diagnostic criteria based on the proportion of elastic fibers detectable by Verhoeff-van Gieson staining in the lesions. For the record, we have the impression that our present case, which we call OEFL, may be closer to what Rodrigues et al. [12] call "oral elastofibroma" than to what they call "oral elastofibroma-like lesion". Their study suggests that the so-called oral fibroma has more elastic fiber growth than previously thought and that OEFL may not be such a rare lesion.

However, none of the 128 cases that we had diagnosed as oral irritation/traumatic fibroma (so-called oral fibroma) from 2010 to 2023 in our departments showed characteristic findings of predominant growth of delicate eosinophilic fibers and absence of marked bundles of collagen fibers, as in the present case. Also in another previous series of 124 cases with 129 oral irritation fibromas [13], there are no lesions that could be diagnosed as OEFL showing such characteristic histopathological findings.

Rodrigues et al. [12] also suggest that the OEF may originate from the OEF-like lesion or the oral fibroma. The OEFL presented by us here may correspond to a special pathological condition that exists in the spectrum from the OEF-like lesion or oral fibroma to the OEF.

The true biological nature of the OEFL, especially whether it is a true tumor, the relationship between the OEFL and extraoral similar lesions including EFd, the relationship between the OEFL and intraoral similar lesions including oral fibroma and the "oral elastofibroma" described by Rodrigues et al. [12], and even whether OEFL is an appropriate name, are unknown at this time. Further accumulation of more cases and large-scale analyses are needed to resolve these issues.

The literature retrieved here provided little information on the previous history of irritant ingestion or contact. However, future accumulation of such information will be important to elucidate the etiology of the OEFL, especially the involvement of such external factors in OEFL, in addition to the history of trauma or surgery at the lesion site. Recent genetic studies of extraoral similar lesions such as EFd have suggested its neoplastic nature, and a similar genetic approach may be needed in the future for the OEFL. However, due to the limited number of cases of OEFL, such an approach is likely to be challenging.

## Conclusions

In this report, we described a case of OEFL involving the tip of the tongue in an 83-year-old male and reviewed the literature regarding the clinicopathological features, differential diagnosis, treatment, and prognosis of this lesion. Although the true biological nature of the OEFL, especially whether it is a true tumor, is unclear at this time, its prognosis is good, with few recurrences after simple resection. Moreover, the OEFL may also be misdiagnosed preoperatively as malignant or precancerous lesions. Therefore, the OEFL should be included in the differential diagnosis when the lesions with such clinicopathologic features are encountered. The Elastica van Gieson staining to detect elastic fibers within the lesions is very useful and significant in the histopathological diagnosis of OEFL. Further studies, especially those with information on the history of irritant ingestion or contact, in addition to the history of trauma or surgery at the lesion site, are needed to elucidate the pathogenesis of the OEFL.
